# Green synthesis and characterization of α-Mn_2_O_3_ nanoparticles for antibacterial activity and efficient visible-light photocatalysis

**DOI:** 10.1038/s41598-024-56666-2

**Published:** 2024-03-21

**Authors:** Saeid Taghavi Fardood, Farzaneh Moradnia, Fateme Yekke Zare, Siamak Heidarzadeh, Mohammad Azad Majedi, Ali Ramazani, Mika Sillanpää, Ky Nguyen

**Affiliations:** 1 Department of Chemistry, Faculty of Science, Ilam University, Ilam, 69315516 Iran; 2 Department of Chemistry, Faculty of Science, University of Zanjan, Zanjan, 45371-38791 Iran; 3 Department of Microbiology and Virology, School of Medicine, Zanjan University of Medical Sciences, Zanjan, Iran; 4 Department of Anesthesiology, Faculty of Medicine, Kurdistan University of Medical Sciences, Sanandaj, Iran; 5 Department of Chemical Engineering, School of Mining, Metallurgy and Chemical Engineering, University of Johannesburg, P. O. Box 17011, Doornfontein, 2028 South Africa; 6 Adnan Kassar School of Business, Lebanese American University, Beirut, Lebanon; 7 Sustainability Cluster, School of Advanced Engineering, UPES, Bidholi, Dehradun, Uttarakhand 248007 India; 8 Centre of Research Impact and Outcome, Chitkara University Institute of Engineering and Technology, Chitkara University, Rajpura, Punjab 140401 India; 9 Department of Civil Engineering, University Centre for Research and Development, Chandigarh University, Gharuan, Mohali, Punjab India; 10 Division of Research and Development, Lovely Professional University, Phagwara, Punjab 144411 India; 11 Institute of Research and Development, Duy Tan University, Da Nang, Vietnam; 12 School of Engineering and Technology, Duy Tan University, Da Nang, Vietnam

**Keywords:** Green synthesis, Manganese (III) oxide, Photocatalysis, Antibacterial, Eriochrome Black T, Photocatalysis, Biosynthesis

## Abstract

In this study, green synthesis, characterizations, photocatalytic performance, and antibacterial applications of α-Mn_2_O_3_ nanoparticles are reported. The synthesized nanoparticles were characterized by Fourier transform infrared spectroscopy (FT-IR), powder X-ray diffraction (XRD), transmission electron microscope (TEM), Scanning electron microscopy (SEM), energy dispersive X-ray analysis (EDX), Brunauer Emmett Teller (BET), Electrochemical Impedance Spectroscopy (EIS), Photoluminescence (PL), and Differential reflectance spectroscopy (DRS) analysis. The investigation verified that the α-Mn_2_O_3_ nanoparticles possessed a cubic structure, with a crystallite size of 23 nm. The SEM and TEM techniques were used to study the nanoscale morphology of α- Mn_2_O_3_ nanoparticles, which were found to be spherical with a size of 30 nm. Moreover, the surface area was obtained as 149.9 m^2^ g^−1^ utilizing BET analysis, and the band gap was determined to be 1.98 eV by DRS analysis. The photocatalysis performance of the α-Mn_2_O_3_ NPs was evaluated for degrading Eriochrome Black T (EBT) dye under visible light and degradation efficiency was 96% in 90 min. The photodegradation mechanism of EBT dye was clarified with the use of radical scavenger agents, and the degradation pathway was confirmed through Liquid Chromatography–Mass Spectrometry (LC–MS) analysis. Additionally, the produced nanoparticles could be extracted from the solution and continued to exhibit photocatalysis even after five repeated runs under the same optimal conditions. Also, the antibacterial activity of green synthesized α-Mn_2_O_3_ nanoparticles was investigated by using the broth microdilution method towards *Enterococcus faecalis* ATCC 29212 (Gram-positive), *Staphylococcus aureus* ATCC 29213 (Gram-positive), *Salmonella typhimurium* ATCC 14028 (Gram-negative), *Klebsiella pneumoniae* ATCC 7881 (Gram-negative), *Escherichia coli* ATCC 25922 (Gram-negative), *Proteus mirabilis* ATCC 7002 (Gram-negative), and *Pseudomonas aeruginosa* ATCC 27853 (Gram-negative) bacterial strains.

## Introduction

With increasing industrial and agricultural activity, the population growth, global climate change, and diminishing water reserves, environmental concern has been aroused. Synthetic dyes are one of the main water pollutants that have been used in various industries^1–3^. Researchers have found that these organic dyes have critical threats for environmental and human health because of their persistence and toxicity. So, water treatment is one of the key concerns of the twenty-first century and finding efficient ways is essential^4–6^. Microbial contamination is another one of the main challenges in food industry and healthcare. Thus, evolvement the antibacterial agents has attracted attention. As reported in the literature, nanoparticles have been used as the useful approach with antibacterial activity^7–10^.

Photocatalysis is a green, effective, and promising way for the degradation of the organic dyes and other pollutants from wastewaters, because it can convert toxic pollutants into non-toxic compounds under light sources (ultra violet, solar, or visible light)^11,12^. Photocatalyst attributes such as band gap, light-harvesting performance, surface area, and morphology have main roles in photocatalytic performance^13–15^. Therefore, developing and synthesizing photocatalyst with high performance is essential for degrading organic dyes in wastewater ^16,17^.

Different methods, such as sol–gel, hydrothermal, sonochemical, microemulsion, co-precipitation, biological synthesis, have been used for nanoparticles synthesis^18–21^. Synthesis of nanoparticles by using plant extracts has attracted attention since it is environmentally friendly, non-toxic, inexpensive, simple, and efficient method^22–26^. In 2020, Chandiran et al. reported the synthesis of α-Mn_2_O_3_ nanorods via the hydrothermal route. They then investigated the effectiveness of these nanorods for the decolorization of methylene blue and rhodamine B dyes as cationic dyes^27^. Gnanam group reported the preparation of alpha manganese sesquioxide (α-Mn_2_O_3_) by hydrothermal method and investigated them for the photodegradation of ye Remazol Red B dye using a multilamp photo reactor^28^.

**Figure 1:**
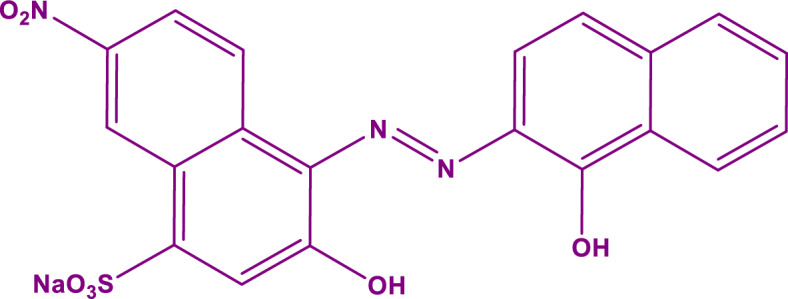
Molecular structure of Eriochrome Black T dye.

In this work, α-Mn_2_O_3_ NPs were prepared with the green sol–gel method by using tragacanth gel (TG), as the natural gel. The synthesized nanoparticles were characterized by FTIR, XRD, DRS, BET, TEM, FESEM, EIS, PL, and EDX analysis. The photocatalytic activity of α-Mn_2_O_3_ NPs was considered for the degradation of Eriochrome Black T (EBT) dye under the visible light irradiation. Moreover, Radical scavenger agents and LC–MS analysis validated EBT dye's photodegradation mechanism. Different Gram-positive bacterial strains such as *Enterococcus faecalis* ATCC 29212, *Staphylococcus aureus* ATCC 29213, and Gram-negative bacterial strains consist of *Escherichia coli* ATCC 25922, *Salmonella typhimurium* ATCC 14028, *Klebsiella pneumoniae* ATCC 7881, *Proteus mirabilis* ATCC 7002, and *Pseudomonas aeruginosa* ATCC 27853 were selected as bacterial models to evaluation the antimicrobial activity of synthesized α-Mn_2_O_3_ nanoparticles. The molecular structure of the EBT dye is shown in Fig. 1.

## Experimental

### Materials and characterization

The tragacanth gum (TG), Eriochrome Black T dye, Mn(NO_3_)_2_.4H_2_O with Purity: ≥ 99%, and bacterial strains were purchased from health food shop, Alvansabet Company (Iran), and Dae-Jung (Korea), Merck (Germany), respectively. The XRD pattern was collected on an X’Pert-PRO advanced diffractometer with Cu-Kα radiation (wavelength: 1.5406 Å) of 40 kV and 40 mA by step scanning in an angle range of 10° ≤ 2θ ≤ 80° at ambient temperature. Using a Metrohm (Analytik Jena-Specord 205) double-beam instrument, UV–Vis absorption spectra were obtained. The Jasco 6300 spectrophotometer recorded the α-Mn_2_O_3_ NPs' FTIR spectra at a resolution of 4 cm^−1^. BET analysis was achieved with the Belsorp Mini II to determine the specific surface area of prepared sample. optical properties were recorded by UV–vis diffuse reflectance spectra (Shimadzu, UV-2550, Japan), and band gap (Eg) was calculated by the Tauc’s theory^29^. TEM was recorded with EM 208S, and SEM coupled by energy dispersive X-ray EDX was investigated with Tescan Mira3. Electrochemical impedance spectroscopy (EIS) was recorded by μ-AUTOLAB electrochemical system type III (Eco-Chemio, Switzerland). The ensuing degradation products of EBT dye were analyzed using a Waters Alliance 2695 HPLC-Micromass Quattro micro API Mass Spectrometer. A Cary Eclipse Fluorescence Spectrometer with a Xe lamp excitation source was used to record room-temperature photoluminescence (PL) spectra.

### Synthesis the α-Mn_2_O_3_ nanoparticles

Firstly, TG solution was prepared based on our previous work^30^. Then 1.5 g of Mn(NO_3_)_2_.4H_2_O were added into the TG solution and the container was moved to the sand bath set at 75 °C, for 12 h under stirring. The dried resin was calcined at 500 °C for 4 h to obtain α-Mn_2_O_3_ nanoparticles.

### Photocatalytic experiment

The photocatalytic performance of α-Mn_2_O_3_ nanoparticles were investigated under visible light with fluorescent lamp (λ > 400, 90 W, Parmis, Iran). The photocatalytic experiments for the degradation of the EBT dye were monitored in 50 ml solution and the effects of visible light irradiation, dark, initial dye concentration (20–50 mg L^−1^) photocatalyst dosage (0.02–0.05 g), time (0–90 min), and *pH* (3, natural, and 9) were studied to find the best degradation efficiency. The degradation efficiency of EBT dye was calculated by UV–Vis spectrophotometer at the λ_max_ = 546 nm.

### Antimicrobial experiment

#### Minimum inhibitory concentration

The minimum inhibitory concentrations (MICs) of α-Mn_2_O_3_ nanoparticle synthesized was evaluated by broth microdilution method against Gram-positive and Gram-negative bacteria. Bacterial strains were separately cultured at 37 °C for 24 h in 5 ml Brain heart infusion (BHI) broth (Merck, Germany). Then, the bacteria were diluted to the 0.5 McFarland standard (1.5 × 10^8^ CFU/ml). Mueller–Hinton broth (200 μl) was added to all 96 microplate wells. The stock concentrations of α-Mn_2_O_3_ nanoparticle were serially diluted to obtain concentrations in the range of 1–5 μg/mL. Approximately 100 μl of α-Mn_2_O_3_ nanoparticle (1–5 μg/mL) was then serially diluted in 1% dimethyl sulfoxide (DMSO) in the 96 wells microplate row-to-row. After that, the prepared bacterial suspension (100 μl) of 0.5 McFarland standard was added to all of the microplate wells and then incubated for 18 h at 37 °C in a shaker incubator at 120 rpm. The MIC was determined as the lowest concentration that inhibited visible bacterial growth observed within the microplate.

#### The minimum bactericidal concentration

The MBC is the minimum concentration of the nanoparticle that is bactericidal. MBC is determined as the lowest broth dilution of α-Mn_2_O_3_ nanoparticle that inhibits growth of the bacteria on the agar plate. The lack of bacterial growth in the Müller-Hinton agar was indicative of MBC.

MBC was measured by sub-culturing the broth dilutions applied for MIC determination on agar media. Briefly, 50 μl of broth dilutions of MIC was sub-culturing onto Müller-Hinton agar (Merck, Germany) and incubated overnight at 37 °C. The lowest broth dilution of nanoparticles that inhibits the growth of bacteria on the agar plate is known as MBC. The lack of bacterial growth in the Mueller–Hinton agar was indicative of MBC.

## Result and discussion

### Characterization

**Figure 2:**
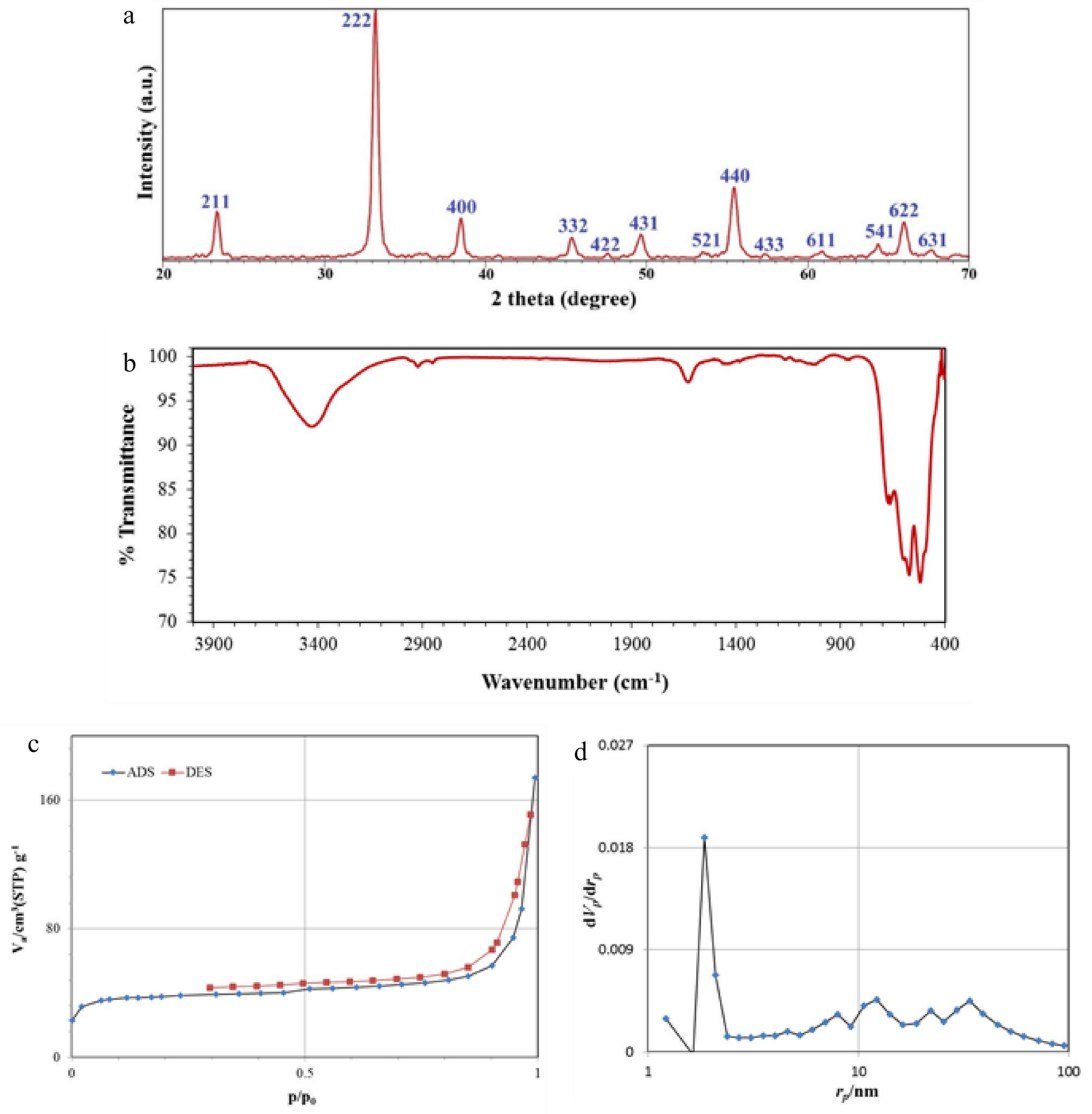
(**a**) XRD pattern, (**b**) FT-IR spectrum, (**c**) The N_2_ absorption/desorption isotherm, (**d**) BJH curve of the α-Mn_2_O_3_ NPs.

Figure 2a demonstrates the XRD pattern of the α-Mn_2_O_3_ nanoparticles. It is evident that sharp and strong diffraction peaks are assigned to cubic structure of synthesized sample (JCPDS card no. 89-4836). Based on the XRD pattern, the crystalline size of α-Mn_2_O_3_ NPs was appraised by Scherrer equation^31,32^ and the obtained value is 23 nm. XRD pattern without characteristic peaks of impurities, confirms the effective synthesis of the α-Mn_2_O_3_ NPs by this green method.

The FTIR spectrum of the synthesized α-Mn_2_O_3_ NPs is given in Fig. 2b. The bands at 3420 and 1624 cm^−1^ corresponds to the O–H stretching and H_2_O bending vibration of the free and absorbed water molecule^29^. Also, the absorption peaks located at 517, 572, 664 cm^−1^ are specified to the vibrational bond of Mn–O, that corroborates successful synthesis of α-Mn_2_O_3_ nanoparticles^28,33^. The nitrogen adsorption–desorption isotherm of α-Mn_2_O_3_ NPs is given in Fig. 2c, and approved the typical type IV with hysteresis loops type H1 based on the IUPAC classification^34^. Moreover, the BET surface area, pore volume, and pore size of sample were found to be 149.9 m^2^ g^−1^, 7.02 nm, and 0.2525 cm^3^ g^−1^, respectively. Also, The BJH curve, which is exposed in Fig. 2d, shows that the pores in α-Mn_2_O_3_ NPs have an average size of 2 nm.

**Figure 3:**
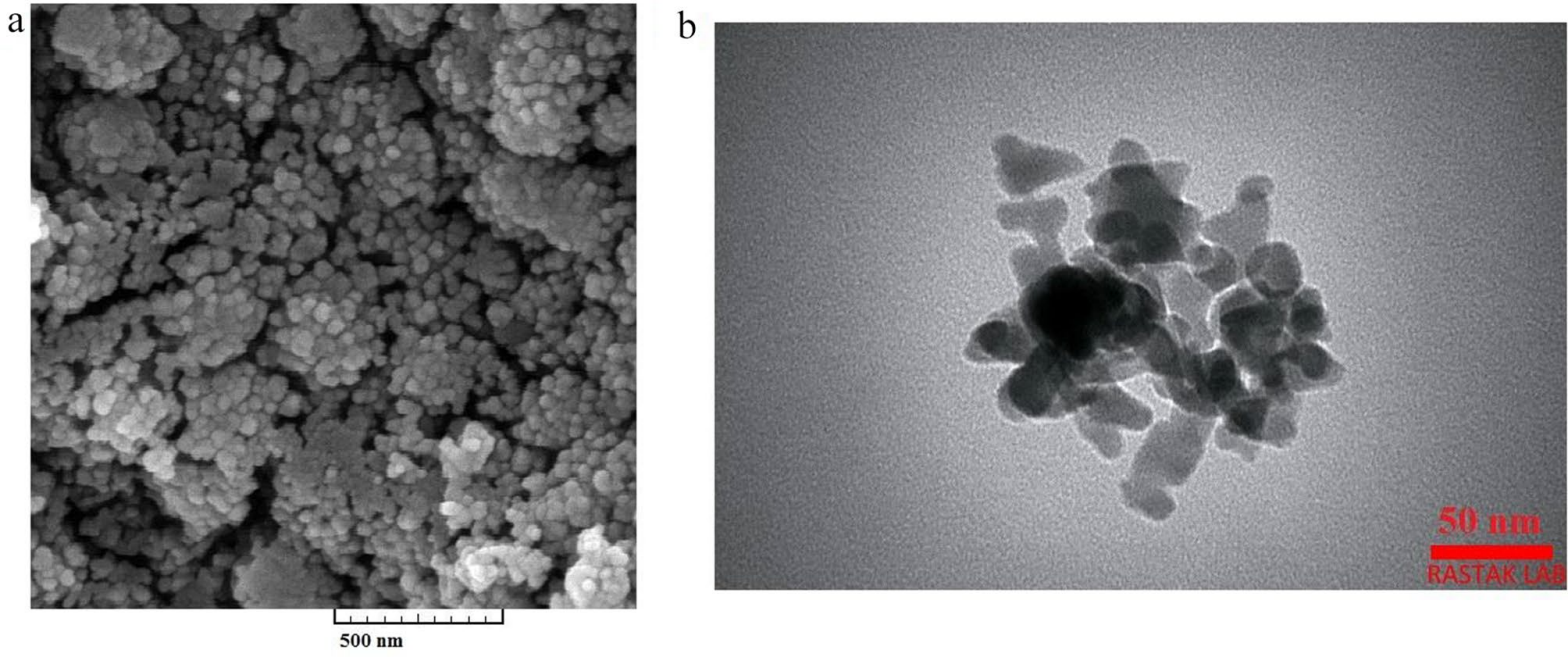
(**a**) FESEM image and (**b)** TEM image of α-Mn_2_O_3_ NPs.

**Figure 4:**
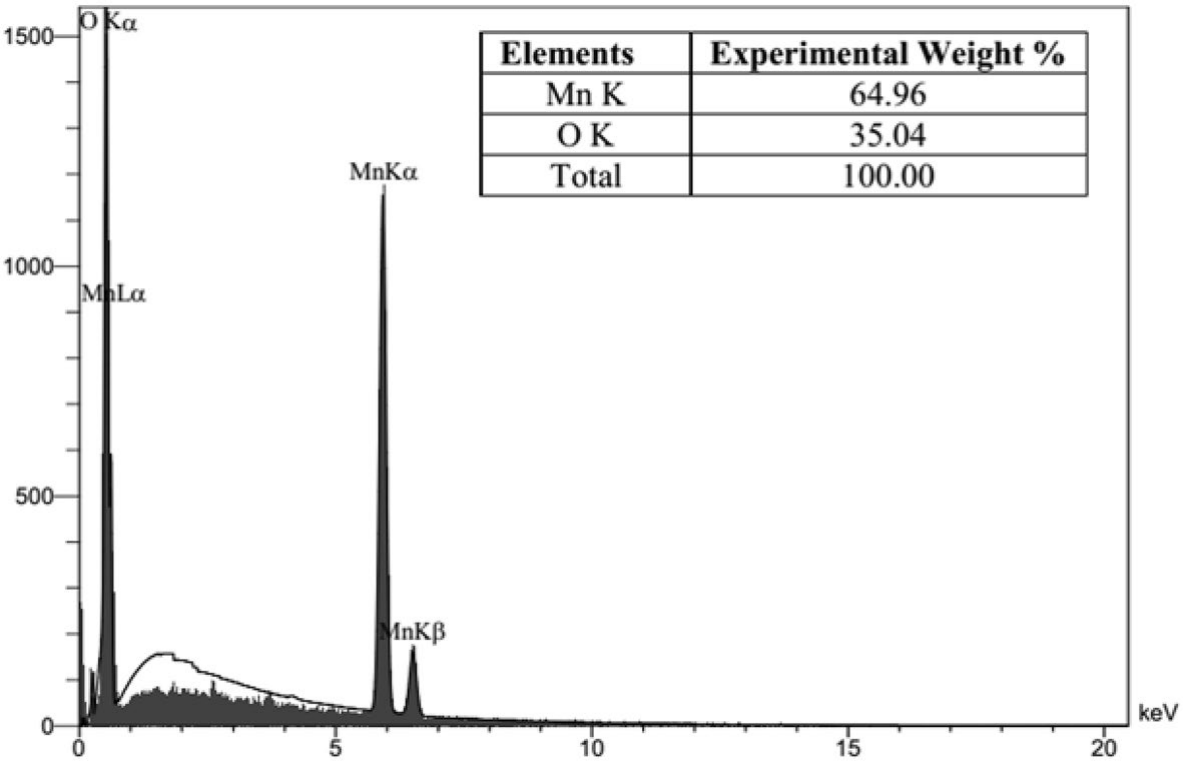
EDX pattern of α-Mn_2_O_3_ NPs.

The TEM and FESEM analysis were carried out to investigate the morphology and size of synthesized nanoparticles and their results are shown in Fig. 3. It is clear that α-Mn_2_O_3_ NPs have a uniform distribution and spherical shape with the average size of 30 nm. Moreover, the EDX pattern confirmed the existence of Manganese (Mn), and oxygen (O) elements in as-prepared sample (Fig. 4).

**Figure 5:**
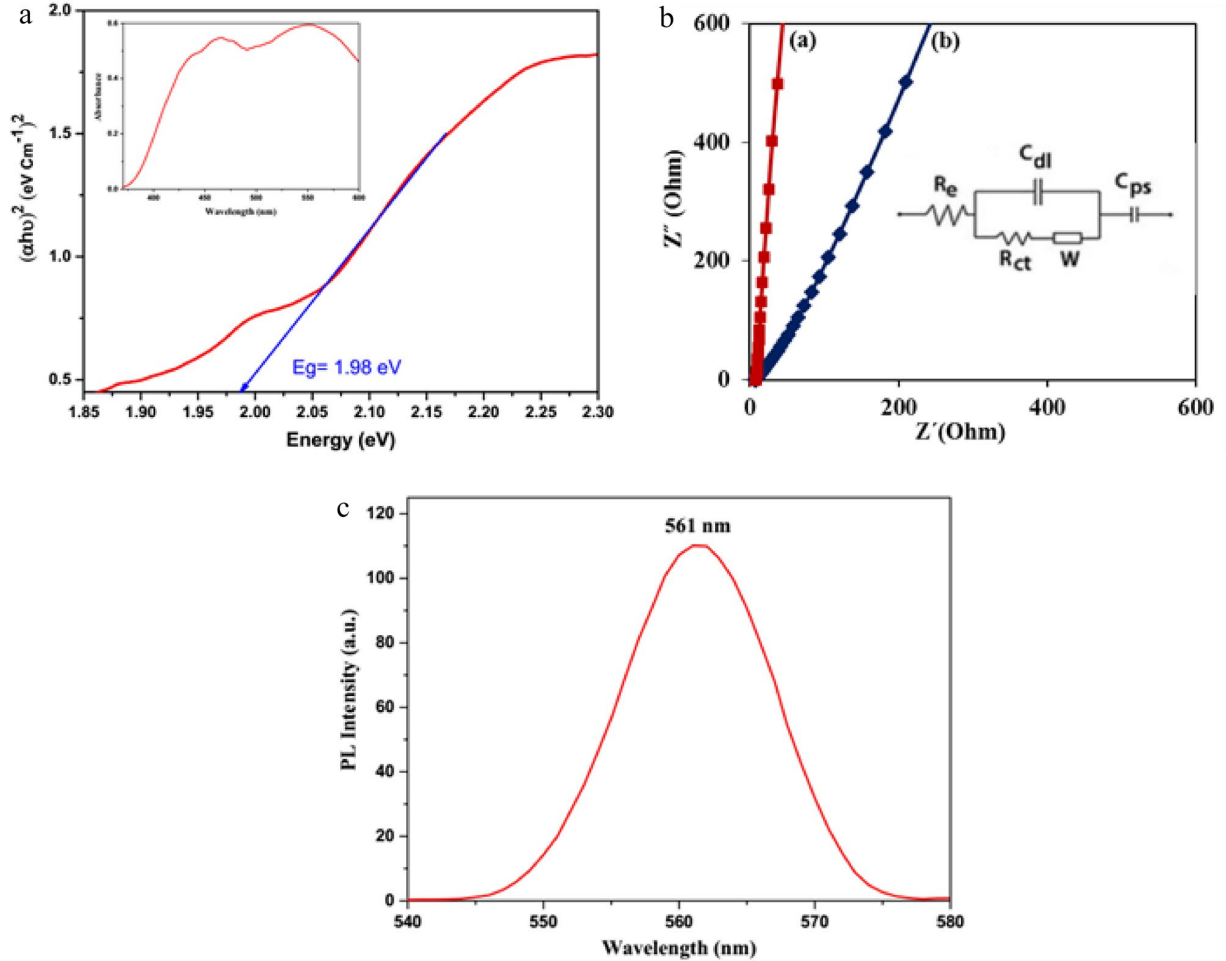
(**a**) UV–Vis-DRS (inset) and Tauc plot, (**b**) the EIS curves of carbon cloth (**a**) and α-Mn_2_O_3_/carbon cloth (**b**) in 1 M KOH and related the equivalent circuit model (inset), (**c**) PL spectra of α-Mn_2_O_3_ NPs.

The UV–Vis-DRS spectrum and tauc plot of the synthesized α-Mn_2_O_3_ NPs are shown in Fig. 5a. Based on the result, the bandgap of sample is about 1.98 eV. This narrow bandgap confirms that synthesized nanoparticles can be suitable for photocatalysis under the visible light irradiation.

EIS was conducted with a 5 mV AC amplitude in the frequency range of 100 kHz–0.1 Hz under open circuit potential. Figure 5b displays Nyquist plots and comparable circuits for bare carbon fabric (a) and α-Mn_2_O_3_/carbon cloth (b). The intersection of the curves at the real portion Z′ (Re) represents a combination of intrinsic resistance of the electrode material, ionic resistance of the electrolyte, and contact resistance at the active material/carbon fabric interface. The Re value for α-Mn_2_O_3_/carbon cloth (7.8 Ω) is comparable to that of bare carbon cloth (6.9 Ω), indicating low intrinsic resistance of the electrode material. Furthermore, α-Mn_2_O_3_/carbon cloth exhibits a low charge-transfer resistance (Rct) of 1.09 Ω, suggesting facile charge-transfer kinetics for the produced α-Mn_2_O_3_ NPs.

Figure 5c shows the room temperature PL spectra of α-Mn_2_O_3_ NPs under laser excitation at 280 nm. The emission at 561 nm is the result of a single ionized oxygen vacancy, which leads to the green emission of the sample due to the recombination of a hole with a single ionized electron in the valence band. This oxygen vacancy is closely associated with the photocatalytic activity of α-Mn_2_O_3_ in degrading EBT. Moreover, numerous studies in the literature have highlighted the role of oxygen vacancies in enhancing photocatalytic reactions^35,36^.

### Photocatalytic activities

To investigate the photocatalytic activity of synthesized sample, α-Mn_2_O_3_ NPs was used in photodegradation experiments toward EBT dyes and obtained results are given in detail.

**Figure 6:**
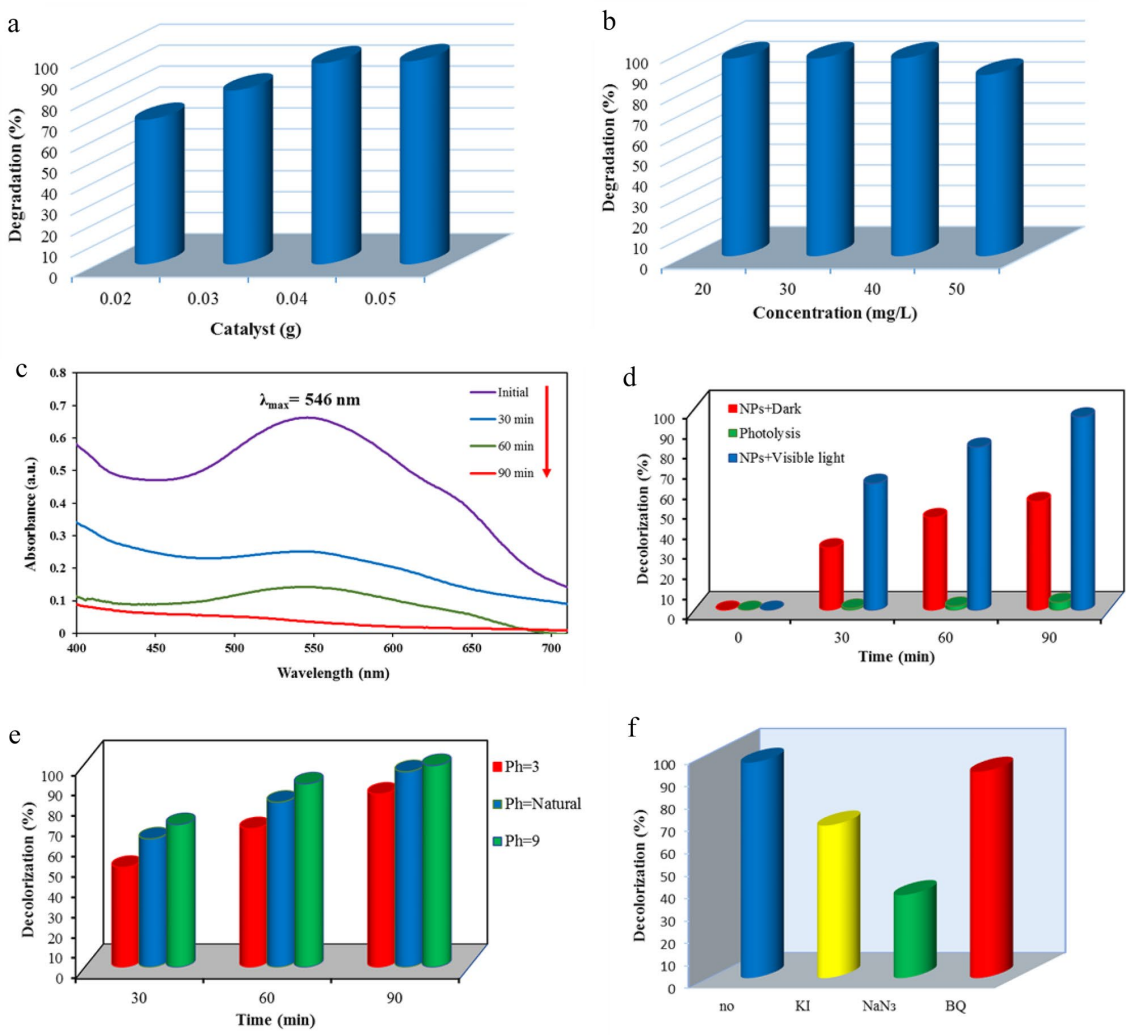
(**a**) The impact of photocatalyst dosage on EBT degradation, *pH* = natural, EBT = 40 mg/L, (**b**) impact of initial concentration on the degradation efficiency (%), *pH* = natural, amount of catalyst = 0.04 g, (**c**) Absorption spectra of EBT solutions under visible light radiation, *pH* = natural, EBT = 40 mg/L, catalyst = 0.04 g. (**d**) Impact of visible light irradiation, *pH* = natural, EBT = 40 mg/L, catalyst = 0.04 g, 90 min, (**e**) impact of *p*H on the degradation efficiency. (**f**) Assessing EBT degradation efficiency in various radical scavenger.

Photocatalyst dosage is one of the important factors in the performance of dye degradation^37^. So, the effect of photocatalyst amount was studied in the range of 0.02–0.05 g of α-Mn_2_O_3_ NPs, at a constant concentration of EBT (40 mg L^−1^) and irradiation time (90 min). As specified in Fig. 6a, the degradation efficiency of EBT raises from 69 to 97% with increasing photocatalysis dosage up from 0.02 to 0.05 g. This may be due to enhance of active sites on the surface of nanoparticles, penetration of visible light radiation into the suspension, and free electrons generation in the conduction band^38,39^. So, next experiments were conducted by using 0.04 g of sample as the best dosage.

To find the effect of initial dye concentration, degradation efficiency was investigated in different concentration of EBT dye (20–50 mg L^−1^). As shown in Fig. 6b, rate of degradation decreased from 96 to 88% in 90 min, when the initial concentration of dye increased from 40 to 50 mg L^−1^. At high concentration, dye molecules inhibit as the penetration and absorption of photons on the surface of the catalyst; for this reason, the degradation rate decreased^37,40^. Thus, the following experiments were conducted by 40 mg L^−1^ of EBT concentration as the best concentration.

To obtain the best irradiation time, the photodegradation study was monitored at diverse time gaps, and obtained result is given in Fig. 6c. The lambda max of EBT dye is placed at 546 nm. Degradation performance was verified with decline in absorbance maximum values while irradiation time increased. It is evident that 96% of EBT dye was removed in 90 min.

Photocatalytic activity of α-Mn_2_O_3_ NPs for dye degradation was proved with checking the efficiency in three states; photolysis (visible light irradiation without α-Mn_2_O_3_ NPs), adsorption (α-Mn_2_O_3_ NPs under dark), and photocatalysis (α-Mn_2_O_3_ NPs under visible light irradiation). In photolysis state, dye degradation was not observed. In adsorption condition, we have dye removal of 54%. As shown in Fig. 6d, in photocatalysis state 96% of EBT dye was removed at 90 min. These results corroborate the high photocatalytic activity of synthesized nanoparticles for EBT degradation under visible light in ambient condition. Figure 6e provides the photodegradation of EBT dye in the presence of α-Mn_2_O_3_ at various *pH* levels and times. Based on the chart, the highest level of degradation occurs at alkaline *p*H. However, significant degradation is also observed at nautral pH, indicating the high activity of the photocatalyst. At higher *p*H values, the concentration of hydroxyl ions increases, resulting in a greater presence of hydroxyl radicals in the medium and consequently faster degradation.

**Figure 7:**
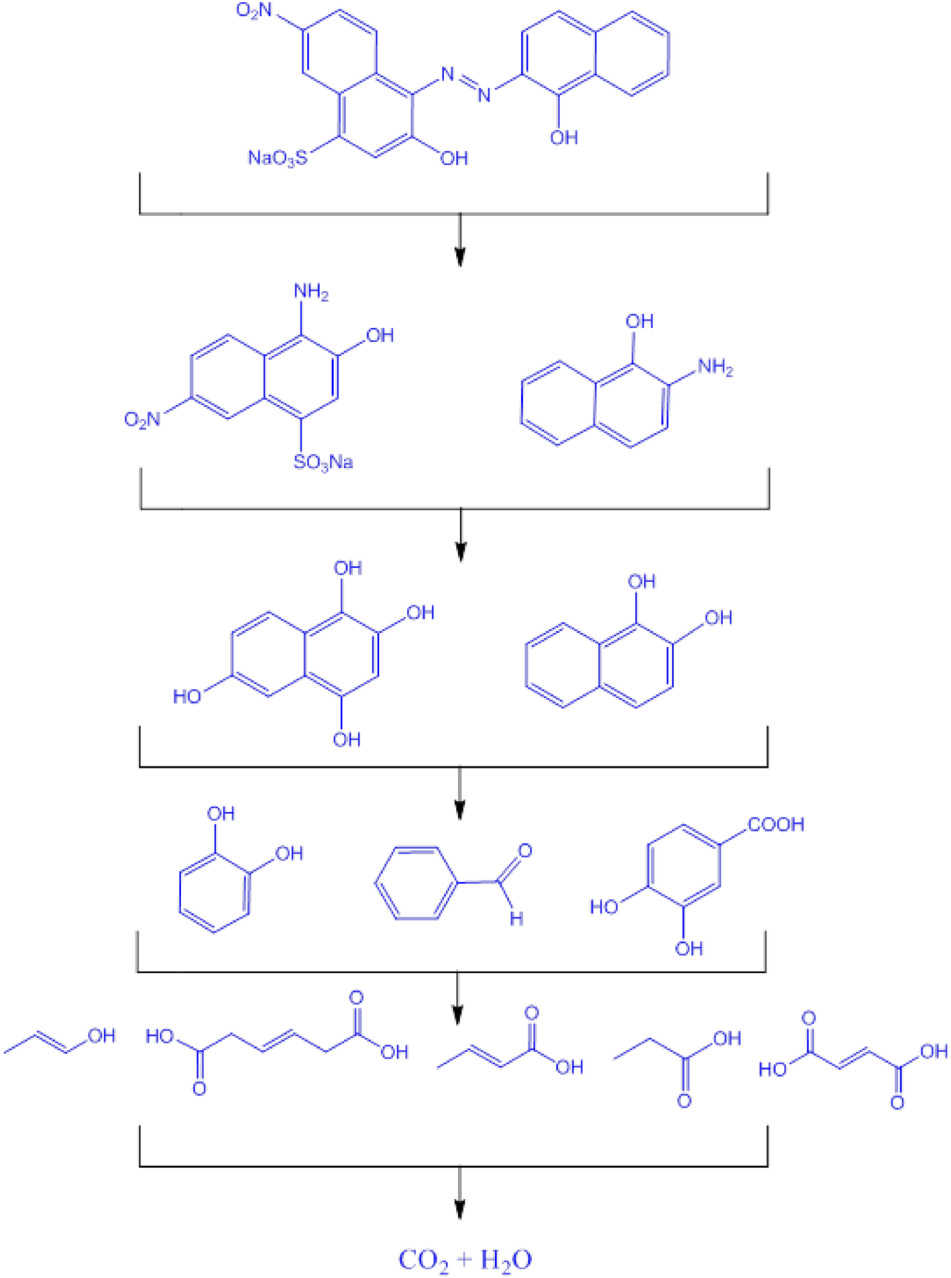
Proposed pathway for EBT degradation using α-Mn_2_O_3_.

To investigate the photodegradation mechanism of EBT dye in the presence of α-Mn_2_O_3_ as the photocatalyst and identify the main reactive species involved, radical trapping studies were conducted (Fig. 6f). The diagram demonstrates that the presence of p-benzoquinone (*p*-BQ), NaN_3_, and KI resulted in a reduction of the degradation efficiency (DE%) from 96 to 92%, 37%, and 68% respectively, after a duration of 90 min. These substances act as radical scavengers for the superoxide radical, singlet oxygen, and hydroxyl radical respectively. It is evident that singlet oxygen has a more significant role in the breakdown mechanism of EBT dye compared to superoxide radical and hydroxyl radical. Figure 7 illustrates the proposed process for the breakdown of EBT dye using LC-Mass analysis. The findings show that there was a breakdown of the EBT dye molecules, leading to the creation of smaller fragmented molecules. Table 1 reports on these broken molecules.

**Table 1:** Identification of by-Products formed during the photocatalytic degradation of EBT.

No	Structure	m/z	No	Structure	m/z
1		58	2		74
3		86	4		115
5		143	6		105
7		110	8		154

**Figure 8:**
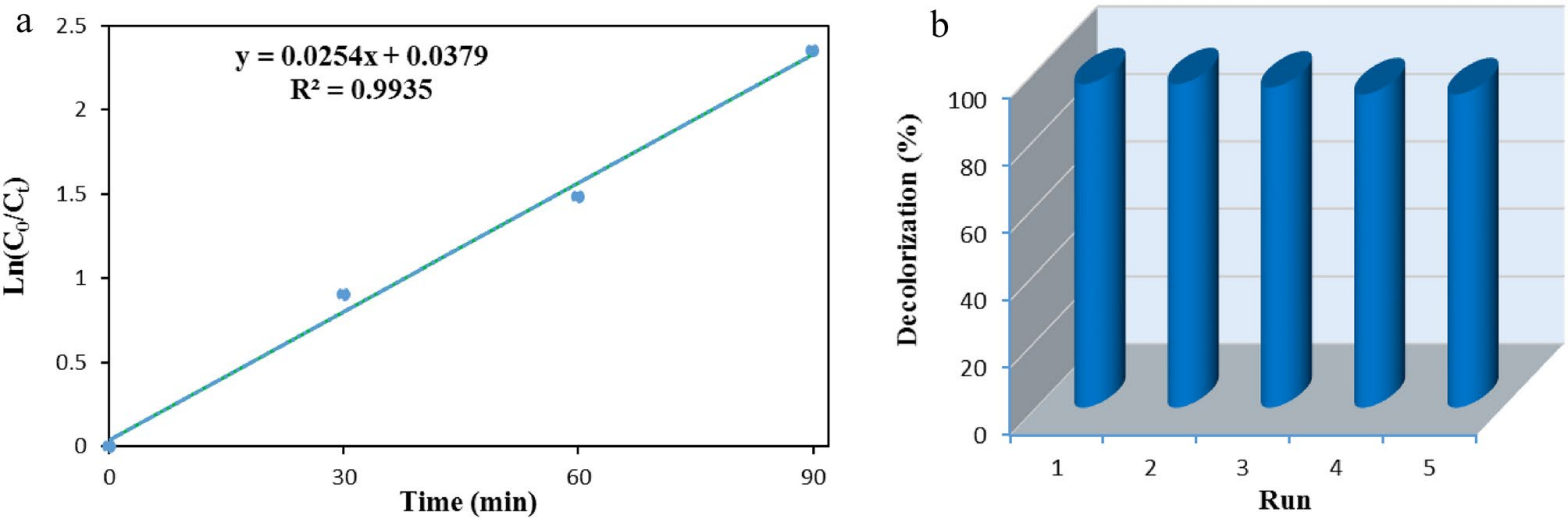
(**a**) Pseudo-first-order kinetic plot for the decolorization of EBT. (**b**) Reusability of α-Mn_2_O_3_ photocatalyst for the decolorization of EBT.

The photodegradation of pollutants often follows first-order reaction kinetics, described by the relationship between the degradation rate and the irradiation time (t) as *ln(C*_*0*_*/C*_*t*_*)* = *kt,* where k is the reaction rate constant^41^. Figure 8a illustrates the kinetics of EBT dye photodegradation by α-Mn_2_O_3_ nanoparticles. The rate constant for EBT dye degradation is *k* = 0.0254 min^−1^, as determined from the slope of the line. Photocatalytic degradation of EBT dye solution can be accomplished by repeated use of the α-Mn_2_O_3_ catalyst. The photocatalytic degradation of EBT under visible light irradiation was measured to assess the reusability of this catalyst. Following centrifugation, washing with distilled water under ultrasonic irradiation, and reusing the photocatalyst for consecutive runs. The photocatalytic efficiency of α-Mn_2_O_3_ nanoparticles experiences a modest decline after undergoing five cycles of use. In Fig. 8b, the photodegradation rates for recycling runs 1–5 were 96%, 96%, 95%, 93%, and 93% respectively. The stability of α-Mn_2_O_3_ NPs was found during the process of photocatalytic oxidation.

The photodegradation capabilities of synthesized α-Mn_2_O_3_ NPs were compared to other photocatalysts for EBT dye degradation to illustrate their efficiency and relevance. Table 2 compares the efficiency of α- Mn_2_O_3_ NPs to different photocatalysts. This work goes beyond method innovation and photocatalytic efficiency to offer significant advantages above earlier publications. Water and tragacanth gum are used to synthesize nanoparticles in an eco-friendly, cost-effective, and simple process. The produced α- Mn_2_O_3_ NPs demonstrate stability, recyclability, and EBT degradation efficiency.

**Table 2:** Photodegradation comparison of EBT with diverse photocatalysts.

Catalysts	Dye concentration (mg/L)	Light source	Irradiation time (min)	Degradation efficiency (%)	Refs
CoCr_2_O_4_	20	Visible light	90	90	^37^
TiO_2_ (PC-50)	25	UV-light	90	35	^42^
TiO_2_ (PC-500)	25	UV-light	90	51	^42^
ZnO (Merck)	25	UV-light	90	62	^42^
TiO_2_	25	UV-light	90	82	^42^
SnO_2_-bentonite	100	UV-light	300	100	^43^
ZnO@Ag_2_S	40	UV-light	120	69	^44^
TiO_2_@CNTs	100	UV-light	100	92	^45^
TiO_2_@CNTs	100	Visible light	100	88	^45^
α- Mn_2_O_3_	40	Visible light	90	96	This study

### Antibacterial activity

The amount of bacteria inhibited by α-Mn_2_O_3_ were variable, among which *S. aureus* and *E. faecalis* (1 μg/mL) were significantly inhibited, and *E. coli*, *K. pneumoniae*, and *P. mirabilis* (2.5 μg/mL) were moderately inhibited. However, *P. aeruginosa* and *S. typhimurium* (3.5 μg/mL) were weakly inhibited by α-Mn_2_O_3_. The highest MBC of α-Mn_2_O_3_ was observed against *S. aureus* and *E. faecalis* (2 μg/mL). The MBC of α-Mn_2_O_3_ against *E. coli* and *P. mirabilis* was 3 μg/mL, and against *K. pneumonia* was 3.5 μg/mL. However, MBC was not detected against *P. aeruginosa* and *S. typhimurium* (Table 3).

**Table 3:** MIC and MBC of α-Mn_2_O_3_ nanoparticle against range of pathogenic bacteria.

Organism	ATCC	Types of bacteria	MIC of α-Mn_2_O_3_ (μg/mL)	MBC of α-Mn_2_O_3_ (μg/mL)	Control (1% DMSO)
*Staphylococcus aureus*	29213	Gram-positive	1	2	Not active
*Enterococcus faecalis*	29212	Gram-positive	1	2	Not active
*Escherichia coli*	25922	Gram-negative	2.5	3	Not active
*Salmonella typhimurium*	14028	Gram-negative	3	Not determined	Not active
*Klebsiella pneumoniae*	7881	Gram-negative	2.5	3.5	Not active
*Proteus mirabilis*	7002	Gram-negative	2.5	3	Not active
*Pseudomonas aeruginosa*	27853	Gram-negative	3	Not determined	Not active

Based on the results, the synthesized nanoparticles exhibited more inhibitory and lethal properties against gram-positive bacteria than gram-negative bacteria, possibly owing to the cellular envelope structure in gram-negative bacteria, because these bacteria have additional outer membrane and porin or porin-like proteins in their envelope structure. The presence of these structures in gram-negative bacteria gives them the ability to selectively enter hydrophobic and hydrophilic molecules inside the bacteria and thus protects the bacterial cell from antibacterial substances.

## Conclusions

The α-Mn_2_O_3_ nanoparticle was successfully prepared by the green and facile sol–gel method without using surfactants and organic solvents. The successful synthesis of the nanoparticles was confirmed by FTIR, XRD, BET, DRS, PL, EIS, TEM, FESEM, and EDX techniques. Based on the XRD and BET analysis, α-Mn_2_O_3_ NPs were prepared in pure cubic structure and high surface area (149.9 m^2^ g^–1^). Also, SEM and TEM images revealed that morphology of nanoparticles is spherical with average width of 30 nm. The photocatalytic activity was verified by comparing the photocatalysis activity with adsorption and photolysis, suggesting a synergistic effect between prepared nanoparticles and visible light toward EBT dye degradation. An analysis of the degradation products of the EBT dye was conducted using LC–MS and proposed pathway for EBT degradation was suggested. The α-Mn_2_O_3_ nanoparticles had appropriate antibacterial activity and showed greater inhibitory and lethal properties against gram-positive bacteria than gram-negative bacteria. This simple method, using renewable tree gum materials and abundant natural resources to prepare the reusable catalyst, would inspire further research into producing and recycling friendly catalysts to degrade different pollutants using abundant light.

## Data Availability

The datasets used and/or analyzed during the current study are available from the corresponding author on reasonable request.
